# Nutritional Optic Neuropathy: Bariatric Surgery Gone Wrong

**DOI:** 10.7759/cureus.84548

**Published:** 2025-05-21

**Authors:** Muhamad Zulhilmi Akmal Zainuddin, Jemaima Che Hamzah, Nik Ritza Kosai Nik Mahmood, Teck Chee Cheng, Mae-Lynn Catherine Bastion

**Affiliations:** 1 Ophthalmology, Universiti Kebangsaan Malaysia, Kuala Lumpur, MYS; 2 Surgery, Universiti Kebangsaan Malaysia, Kuala Lumpur, MYS

**Keywords:** bariatric surgery, central scotoma, copper deficiency, morbid obesity, nutritional optic neuropathy, optic neuropathy

## Abstract

A 52-year-old woman presented with a two-week history of progressive and painless bilateral central blurring of vision, associated with malaise and loss of appetite. Bariatric surgery was done 18 months prior, which was complicated by malnutrition. Ophthalmic examination showed reduced visual acuity and color vision (red-green color vision deficiency) bilaterally with no relative afferent pupillary defect. Examinations of the anterior segment and fundus were unremarkable. A Humphreys visual field test revealed bilateral central scotoma. Optical coherence tomography of the retinal nerve fiber layer and macula was normal. Laboratory investigations showed low serum copper levels (50.29 ug/dL, normal: 80-155 ug/dL). Serum iron, vitamin B12, and folate were normal. Magnetic resonance imaging of the brain and orbit showed no abnormalities. A diagnosis of nutritional optic neuropathy was made. The patient was given total parenteral nutrition (including copper) and oral multivitamin supplements. Over time, her bilateral vision improved to 6/6 N5, and her color vision normalized. Unfortunately, the bilateral central scotoma persisted up to 18 months.

## Introduction

Bariatric surgery (BS) is a gastrointestinal surgical procedure performed in patients who are morbidly obese and do not respond to conservative treatment. This procedure aims to reduce the patient’s body weight through limited gastrointestinal absorption of nutrients or reduction of dietary intake [1-3]. BS can be categorized as restrictive, malabsorptive, or both [4]. Restrictive BS includes laparoscopic adjustable gastric banding and laparoscopic sleeve gastrectomy (LSG). Malabsorptive BS includes biliopancreatic diversion and duodenal switch. Finally, Roux-en-Y gastric bypass (RYGB) is both a restrictive and malabsorptive procedure.

Despite the benefit of BS in reducing body weight, it is associated with a higher risk of malnutrition postoperatively. Anatomical reorganization that occurs due to BS is associated with significant risks of nutrient malabsorption, such as with vitamin B1, B6, B12, folic acid (B9), vitamin D, vitamin E, zinc, and copper [1,5]. These nutrient deficiencies will lead to unfavorable effects on neural tissue function, with nutritional optic neuropathy (NON) being one such complication.

BS is routinely performed because it is a safe and effective treatment for weight loss in obese individuals, with NON being a rare complication [3]. Postoperative care includes long-term or lifelong nutrient supplementation and regular monitoring of micronutrient levels [4,6]. The number of BSs performed has increased globally due to rising rates of morbid obesity.

Herein, we present the case of a patient with NON, which developed less than two years after receiving BS, complicated by malnutrition. We aim to highlight ophthalmic complications that can occur after BS so that they can be avoided. This article was previously presented as a poster at the 12th Conjoint Ophthalmology Scientific Conference on 15-17 September 2023.

## Case presentation

A 52-year-old woman with a history of BS (gastric bypass) presented to the Ophthalmology Department of Hospital Canselor Tuanku Muhriz Universiti Kebangsaan Malaysia with a two-week history of bilateral blurring of vision. The blurring of vision was described as centrally located, and it was progressive in nature. The patient denied any eye pain or eye redness. There were no floaters or flashes of light. She denied any trauma to the eyes, and she had no history of eye surgery. Apart from the bilateral blurring of vision, she also complained of severe malaise and loss of appetite during her presentation. Upon further review of her medical history, it was found that BS was done 18 months prior, which was complicated by malnutrition.

An ophthalmic examination was performed at presentation, wherein her right-eye visual acuity was 6/30 pinhole 6/12 N48, and her left-eye visual acuity was 6/12 pinhole 6/9 N48. Red desaturation was present, as well as reduced color vision bilaterally. However, there was no relative afferent pupillary defect. Anterior segment examination over both eyes was unremarkable. Both eyes showed white conjunctivae with a clear cornea. The anterior chamber was deep and quiet. Her intraocular pressure was 10 mmHg for the right eye and 12 mmHg for the left eye. Her lenses were clear. Fundus examinations were also unremarkable for both eyes. Both optic discs were pink and not swollen or hyperemic. For both eyes, her cup-to-disc ratios were 0.5, and her maculae and retinas were normal.

**Figure 1:**
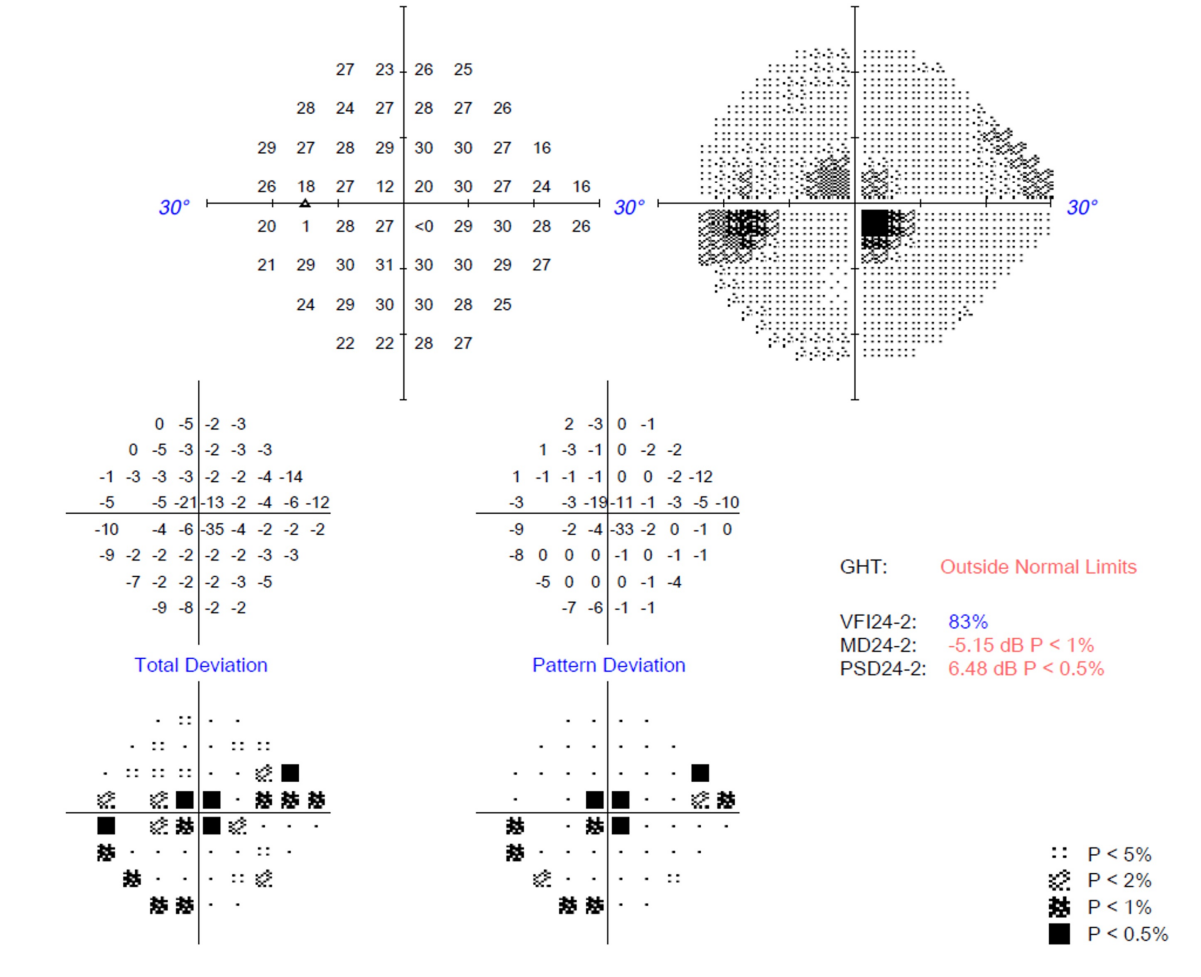
Humphrey visual field (HVF) 24-2 perimetry test over the left eye upon initial presentation showing central scotoma.

**Figure 2:**
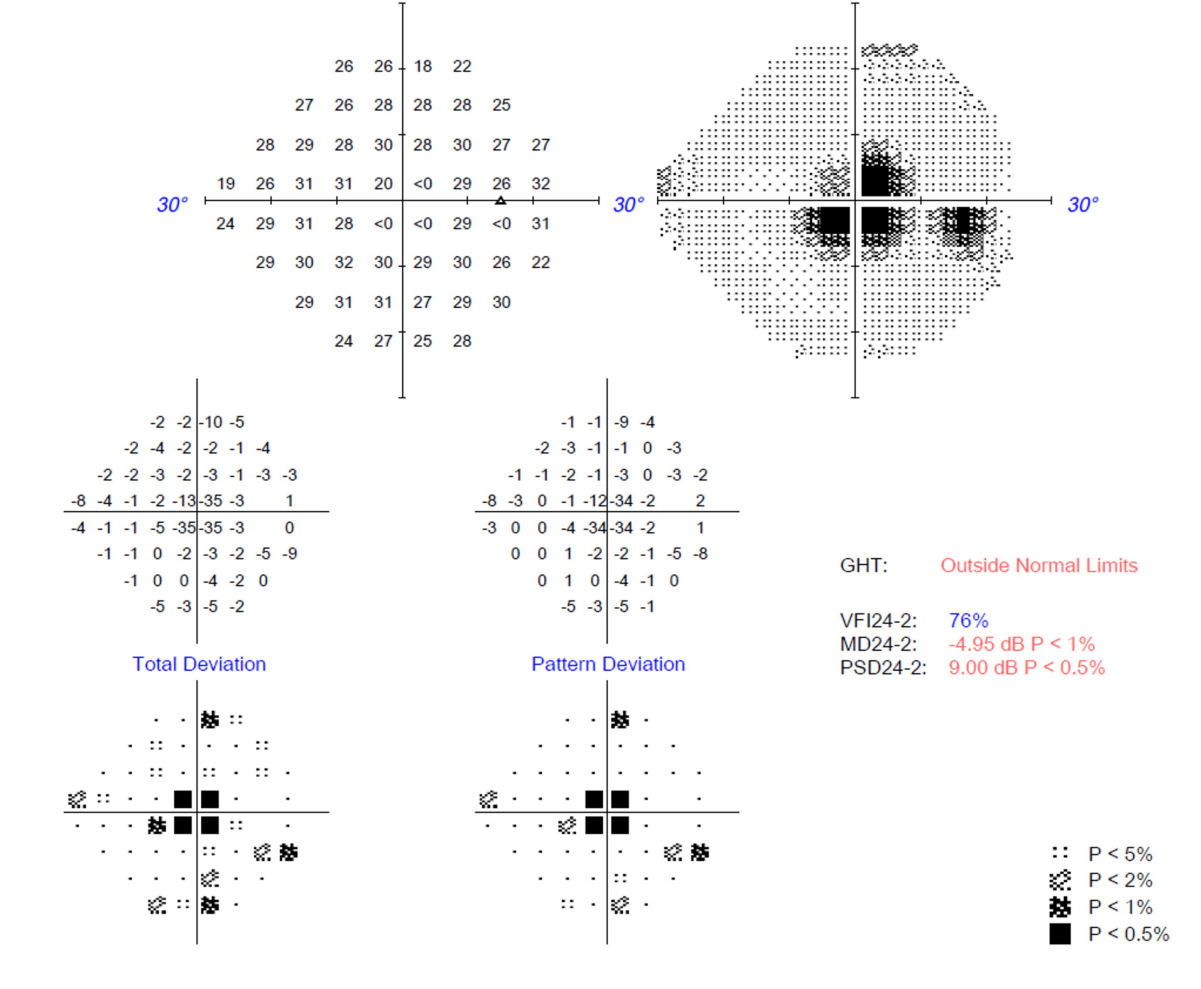
Humphrey visual field (HVF) 24-2 perimetry test over the right eye upon initial presentation showing central scotoma.

**Figure 3:**
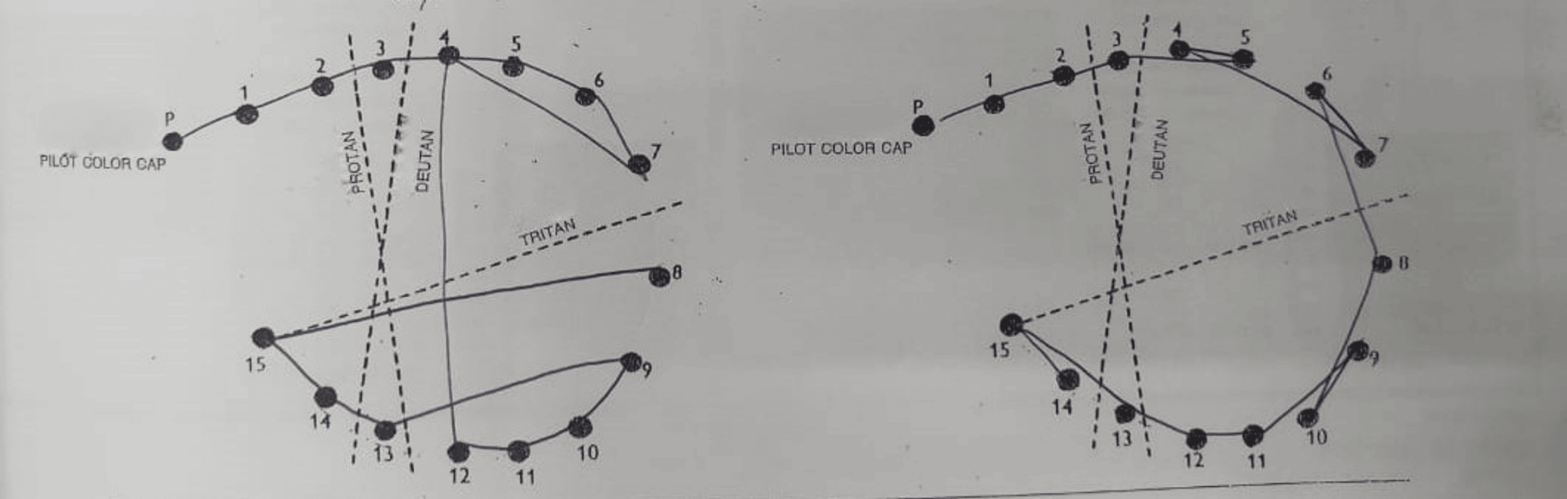
Fransworth Dichotomous D15 color vision test showing abnormal results over both eyes.

**Figure 4:**
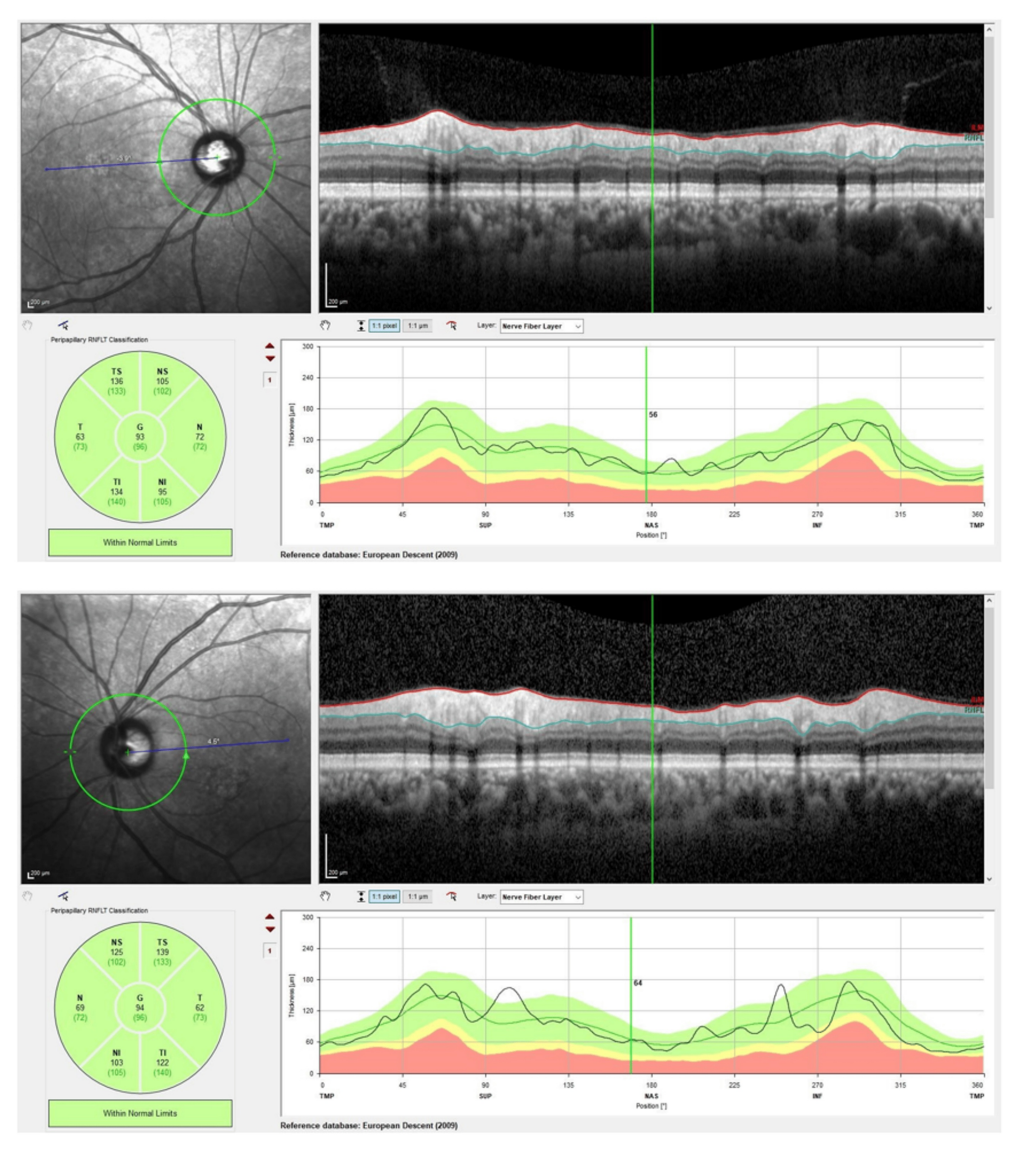
Optical coherence tomography (OCT) of the retina nerve fiber layer (RNFL) over bilateral eyes upon initial presentation showing normal result.

**Figure 5:**
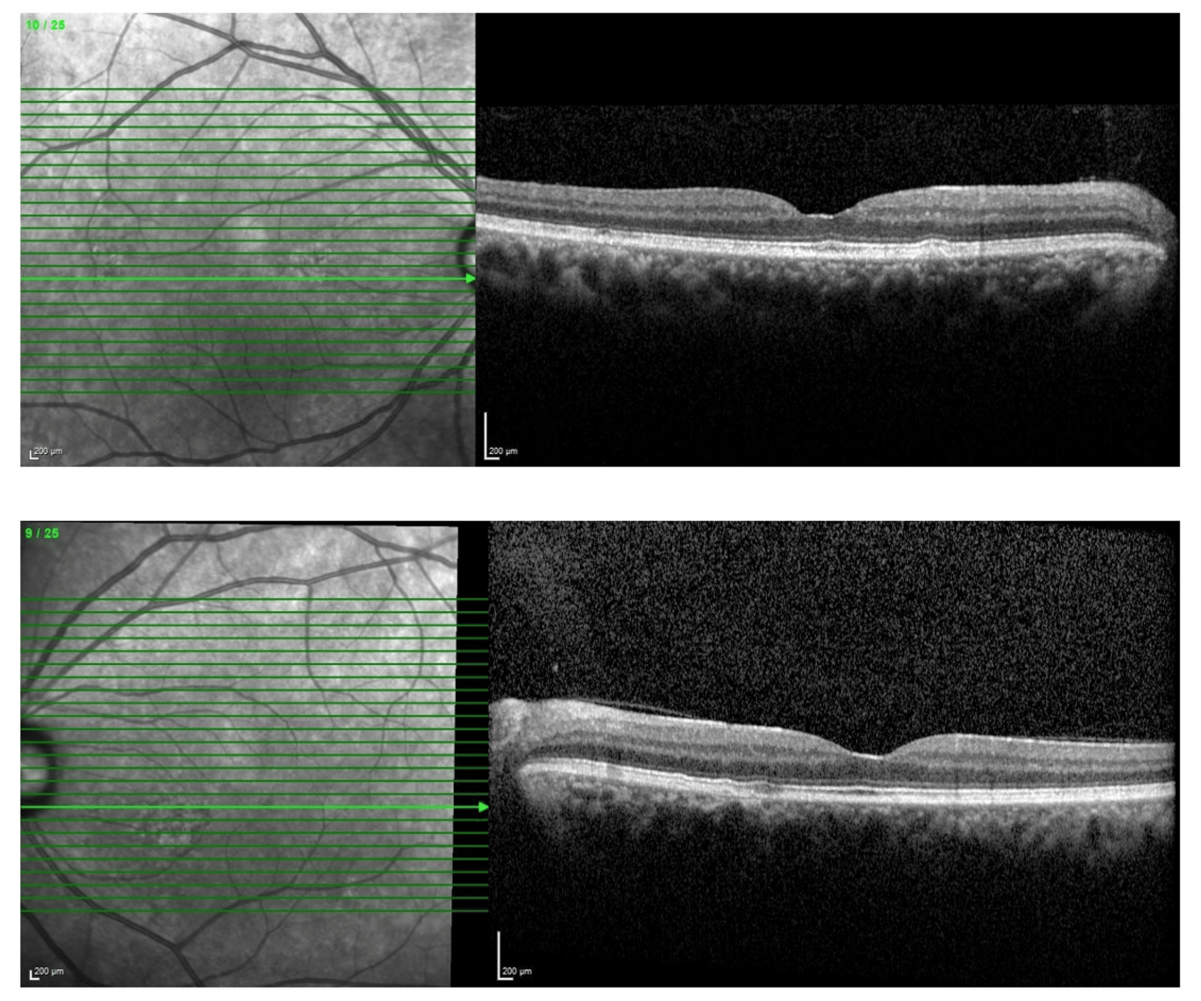
Optical coherence tomography (OCT) of the macula over bilateral eyes upon initial presentation showing no abnormality.

Several ocular investigations were conducted. A Humphrey visual field perimetry test revealed the presence of bilateral central scotoma (Figures 1, 2). In addition, a color vision test (Fransworth Dichotomous D15 test) was performed, and the results were abnormal (Figure 3). Optical coherence tomography (OCT) of the retinal nerve fiber layer (RNFL) and macula was also performed and noted to be normal (Figures 4, 5).

Further evaluations were conducted, including laboratory testing and diagnostic imaging. Laboratory investigations for micronutrients and vitamin levels showed a low serum copper level of 50.29 ug/dL (normal range: 80-155 ug/dL). Meanwhile, serum iron and serum folate (B9) were normal (13.2 umol/L and 36.0 nmol/L, respectively), and serum vitamin B12 was high (3045 pmol/L), as seen in Table 1. The patient’s serum vitamin B12 level was high, as she had a history of admission one month prior for Wernicke encephalopathy, where she was treated with intravenous parentrovite (containing high doses of vitamin B and C) and tablet neurobion.

**Table 1:** Serum micronutrient and vitamin levels.

Investigation	Result	Normal range
Serum copper	50.29 ug/dL	80–155 ug/dL
Serum iron	13.2 umol/L	9.0–30.4 umol/L
Serum folate (B9)	36.0 nmol/L	7.0–46.4 nmol/L
Serum vitamin B12	3045 pmol/L	138–652 pmol/L

Magnetic resonance imaging (MRI) of the brain and orbit revealed no pathologies of the visual pathway. There was no focal enhancing brain parenchymal lesion, and the bilateral optic nerves were not thickened, with preserved signal intensity. The optic chiasm was also normal. Based on the results of the examinations and investigations, a diagnosis of bilateral NON associated with copper deficiency was made.

For treatment, the patient was co-managed with the surgical department team, and she was admitted to the hospital. Throughout her admission, she was given total parenteral nutrition, which included copper and oral multivitamin supplements (i.e., methylcobalamin, neurobion, and folic acid). Although the central scotoma was likely caused by optic nerve pathology, Vitalex was also given if there was maculopathy caused by nutritional deficiency, which was subclinical. The patient also underwent urgent revision of her gastric bypass to its normal anatomy due to severe nutritional deficiency.

**Figure 6:**
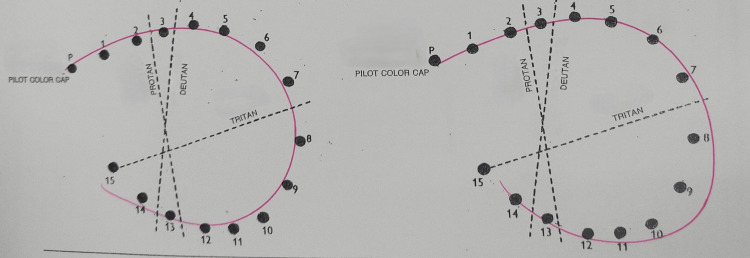
Fransworth Dichotomous D15 color vision test after two months post-treatment showing normalization of bilateral color vision.

**Figure 7:**
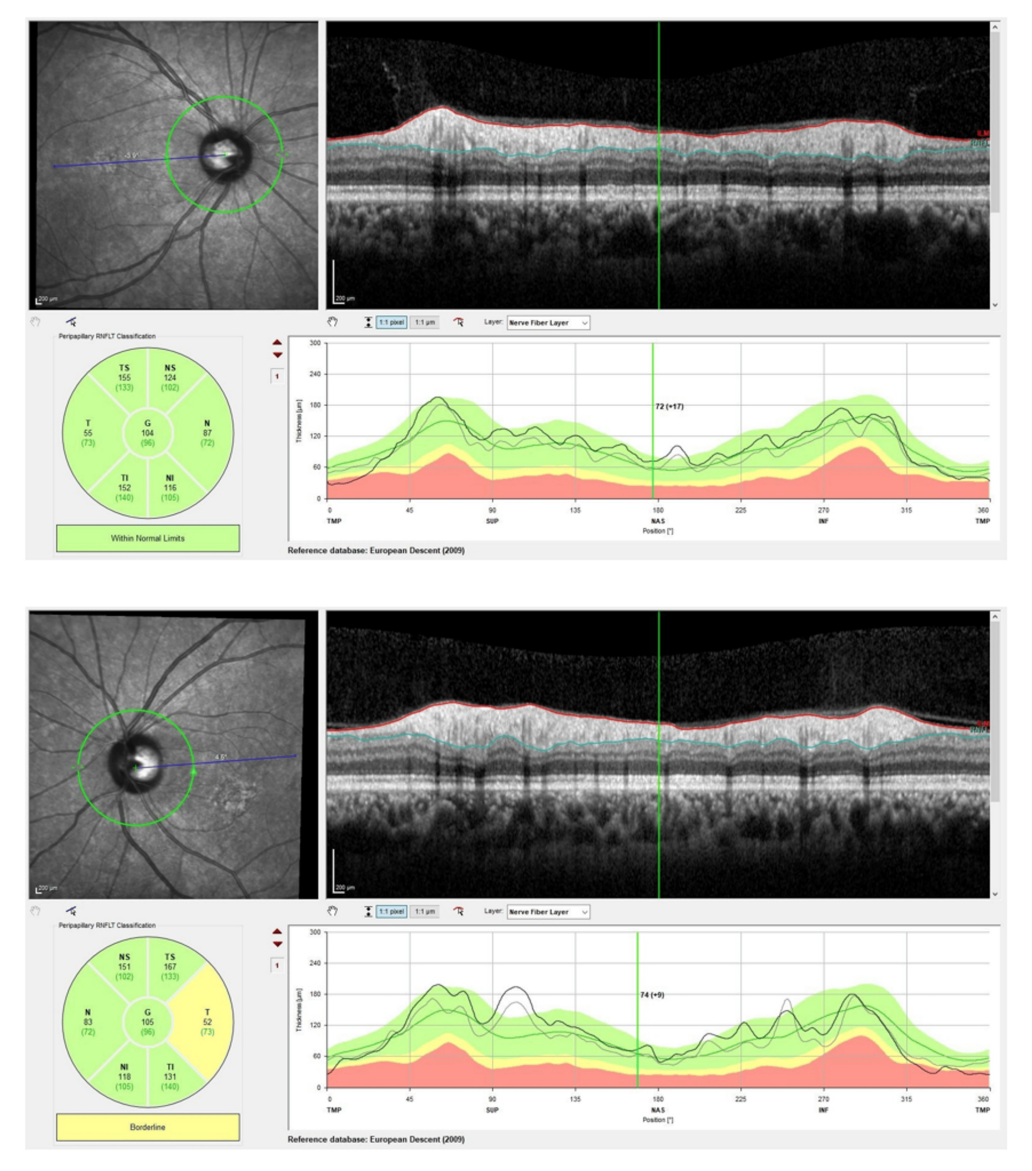
Optical coherence tomography (OCT) of the retina nerve fiber layer (RNFL) over bilateral eyes after four months post-treatment showing borderline thinning over the temporal neuroretinal rim of the left eye. Right eye RNFL was normal.

**Figure 8:**
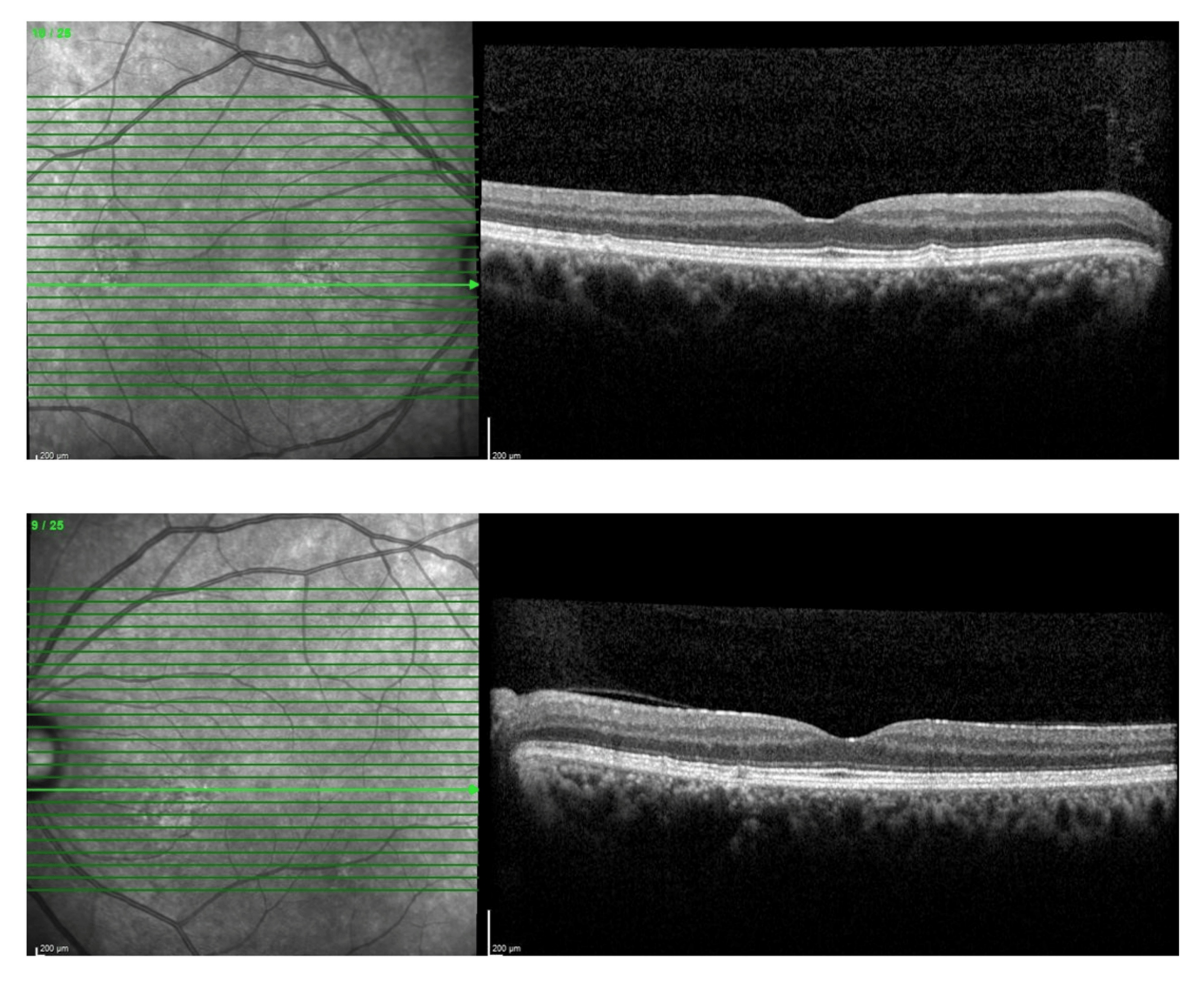
Optical coherence tomography (OCT) of the macula over bilateral eyes after four months post-treatment showing no abnormality.

**Figure 9:**
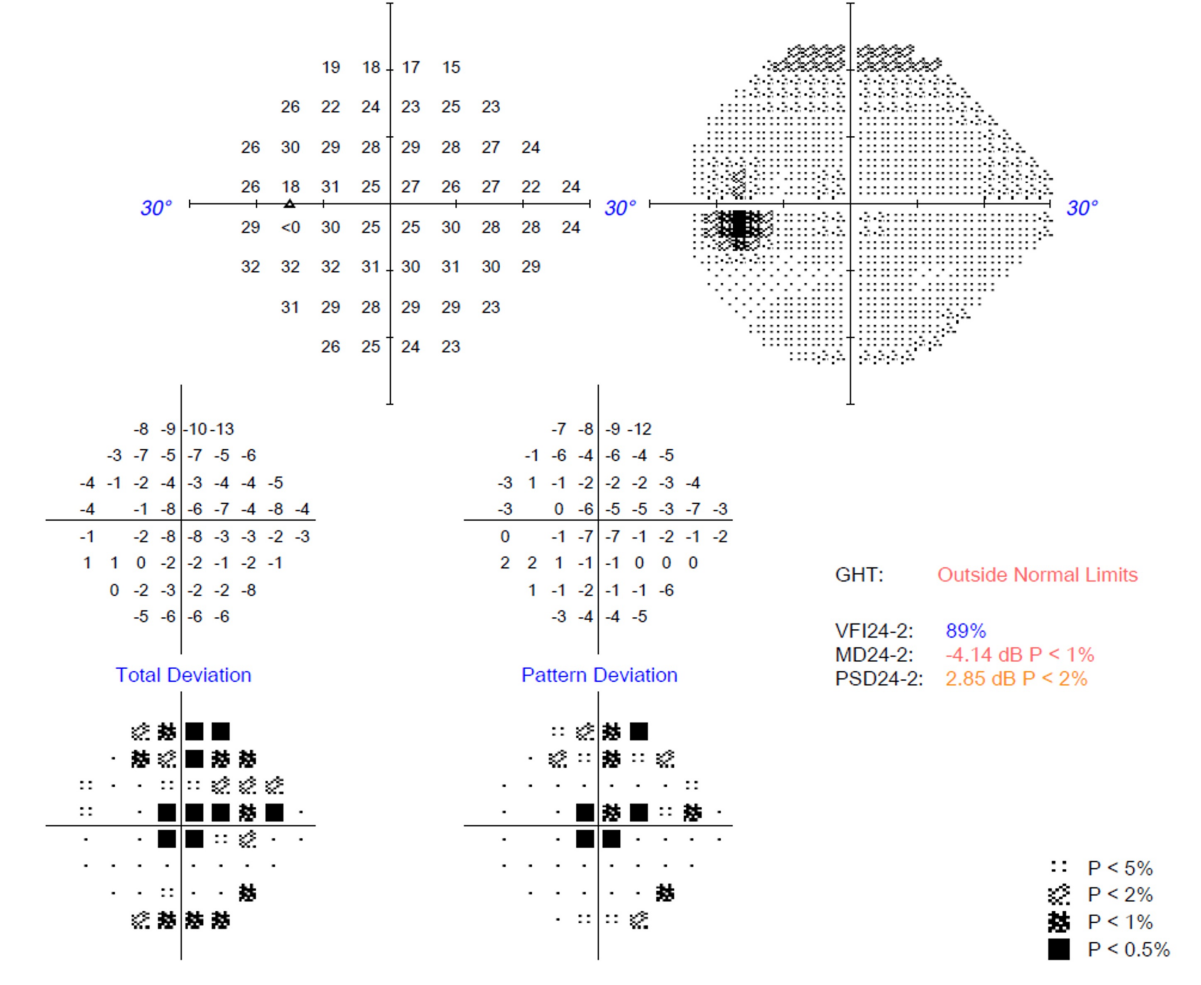
Humphrey visual field (HVF) 24-2 over the left eye after 18 months post-treatment showing persistent central scotoma.

**Figure 10:**
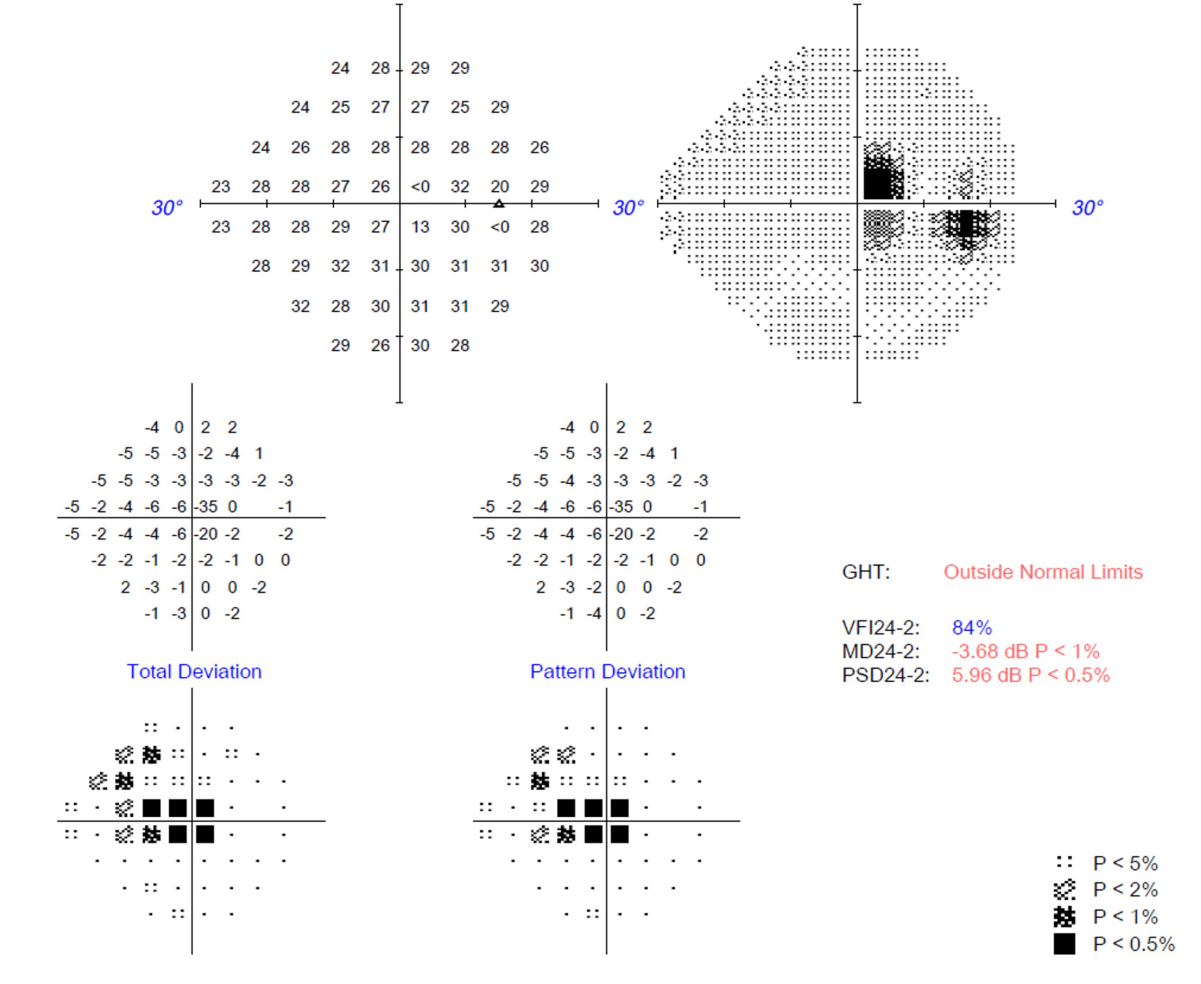
Humphrey visual field (HVF) 24-2 over the right eye after 18 months post-treatment showing persistent central scotoma.

Over time, her bilateral vision improved to 6/6 N5, and her color vision normalized bilaterally (Figure 6). Her bilateral far vision and color vision improved after two months. However, there was a markedly slow recovery for her bilateral near vision, which improved six months later. OCT of the RNFL and macula over bilateral eyes was performed after four months of treatment (Figures 7, 8). There was borderline thinning over the temporal neuroretinal rim of the left eye, whereas for the right eye, the RNFL was normal. On the other hand, OCT of the macula for both eyes was noted to be normal. Unfortunately, her bilateral central scotoma persisted even after 18 months of follow-up (Figures 9, 10).

## Discussion

NON is part of a spectrum of metabolic neuropathies, and it is a rare cause of visual loss. It is characterized by mitochondrial damage secondary to lack of some important nutrients needed for their function, such as copper and vitamin B groups of importance like B12 (cyanocobalamin), B1 (thiamine), and B9 (folic acid). These nutrient deficiencies impair mitochondrial metabolism by interrupting electron transport and subsequently causing a reduction of ATP production, causing poor cell vitality [7]. Thus, such deficiencies lead to mitochondrial dysfunction and oxidative stress, causing neuronal injury [2].

These micronutrients play an important role as coenzymes in mitochondrial energy production in the optic nerve, especially the papillomacular bundle. They are involved in the pathways of carbohydrate, lipid, and nucleic acid metabolism, which are essential for axonal maintenance and myelin synthesis [8]. In NON, there is typically a preferential loss of the papillomacular bundle fibres. This is attributed to the small caliber of the parvocellular retinal ganglion cells located in the papillomacular bundle. They have more limited mitochondrial energy reserves compared to the larger caliber of the magnocellular retinal ganglion cells. This will lead to a lower threshold for apoptosis of these small cells, leading to axonal degeneration [7]. As shown in our case, the patient developed a persistent central scotoma post-treatment despite improvement of visual acuity and color vision. This is likely secondary to the irreversible axonal loss in the papillomacular bundle region.

NON usually presents bilaterally in the eyes with symmetrical involvement. It is progressive, and patients usually develop painless visual impairment with loss of central visual acuity, reduced contrast sensitivity, dyschromatopsia, and a central or centrocecal scotoma [7,9]. Relative afferent pupillary defect is usually not present, as the pupil involvement is bilateral and symmetrical. During the initial phase, the optic disc has no clinical changes, such as in this patient, but subsequently, the optic discs can appear pale, swollen, or hyperemic later on. In the final stages of disease, permanent optic atrophy may appear [7]. Initially, it was unclear whether the patient had a reversible loss of vision, color vision, and visual field. In addition, it became apparent that her vision recovery seemed to lag behind the restoration of nutritional status.

To rule out dyschromatopsia, color vision tests such as the Ishihara test or Fransworth D15 test are important. In our patient, the results of her color vision test were abnormal, which supported optic neuropathy by selective loss of red and green color vision. Ocular investigations such as the Humphrey visual field perimetry test can be done to demonstrate symmetrical central or centrocecal scotoma, which was detected in our patient even until her final review. Optical coherence tomography (OCT) of the optic nerve head and RNFL is another important tool that can help to detect RNFL thinning-even before fundus changes appear. In this case, RNFL was normal at presentation for both eyes, which is consistent with known literature on optic neuropathies [10]. The normal RNFL thickness initially noted supported a reversible condition, given that optic atrophy usually involves ganglion cell loss.

However, according to Kupersmith et al. [11], RNFL thinning is not typically detected until three months after disease onset. Therefore, RNFL thickness typically will be normal during presentation despite the presence of visual loss, indicating optic neuropathy. Similarly, in our case, only at four months post-treatment, OCT of the RNFL started to show borderline thinning over the temporal neuroretinal rim of the left eye, which indicates papillomacular bundle involvement. According to Roda et al. [7], RNFL thinning in NON begins in the papillomacular bundle and then involves all the quadrants. Damage to the papillomacular bundle, which carries signals from the macula, will lead to central scotoma in which correlates with the persistent central scotoma in this case. The poor overall condition of our patient in the preceding months suggested a prolonged state of deficiency.

OCT of the macula can be performed to exclude any anatomical retinal or macular disease, but it is not able to prove macula dysfunction. In such cases, a multifocal electroretinogram (ERG) is useful for detecting dysfunction of the macula. However, our center only had a standard ERG. Visual evoked potential (VEP) is another adjunctive test that can be performed. VEP is useful in assessing the overall electrophysiologic function of the visual pathways, especially in cases with a normal fundus appearance despite significant visual loss [12]. VEP may detect normal or near normal latency with significantly reduced amplitude in NON, even when OCT appears normal [7]. This supports early functional optic pathway dysfunction and may help in equivocal cases. Apart from ocular investigations, imaging studies such as MRI of the brain and orbit are performed to exclude any compressive or demyelinating lesions [7]. In a case of suspected NON, it is important to determine serum levels of micronutrients to identify the underlying deficiencies. Although the other B vitamin levels (B1 and B6) were ordered, the results could not be traced. Regardless, the primary team quickly replaced all micronutrients before blood test results to prevent further systemic damage.

According to Spinazzi [12], the diagnosis of NON is based on three criteria. The first criterion is the exclusion of alternate diagnoses, which includes both inflammatory (optic neuritis) and non-inflammatory optic neuropathies. The second criterion is biochemical detection of deficiencies of micronutrients known to cause optic neuropathy in a patient with a consistent clinical/instrumental presentation. The third criterion is a positive response (clinical and instrumental) to supplementation therapy. Our patient fulfilled these criteria, but there were some missing results, and her response to therapy was slow in terms of her near vision.

BS is an effective treatment for morbidly obese patients who fail to respond to conservative treatment, as it improves metabolic disorders and comorbidities associated with obesity. However, despite these benefits, BS is associated with a high risk of malnutrition postoperatively. Malnutrition complications that occur after BS can be classified into two categories: macronutrient deficiencies and micronutrient deficiencies. For macronutrient deficiencies, proteins and fats are usually affected. Micronutrient deficiencies can involve many vitamins, minerals, and trace elements such as water-soluble vitamins (B1, B2, B3, B5, B6, B7, B9, B12, C), fat-soluble vitamins (A, D, E, K), iron, calcium, iodine, zinc, copper, selenium, chromium, and manganese. Among all BS procedures, the most commonly performed are LSG and RYGB. LSG carries a smaller risk for nutritional deficiencies, whereas gastric bypass is associated with increased risk for nutritional deficiencies [13]. This is because the gastric bypass procedure is more complex, and it changes the gastrointestinal anatomy. In our case, the patient underwent gastric bypass surgery and presented with bilateral NON associated with copper deficiency.

Optic neuropathy post-BS usually presents 18 months to three years postoperatively [2,14] and is associated with vitamin B12, folic acid, or copper deficiency [5], as illustrated by our patient. However, the timing of presentation can vary according to the nutrient deficiencies responsible [9]. For example, in vitamin B12 deficiency, symptoms can occur after several months to a year. In contrast, in copper deficiency, symptoms typically appear after more than three years post-operatively. The present case showed an example of such complications associated with copper deficiency. Eighteen months after BS (gastric bypass) complicated by malnutrition, the patient developed bilateral NON associated with copper deficiency. The timing of presentation for this case is atypical, as it occurred earlier than three years postoperatively.

As copper is a mineral micronutrient that is primarily absorbed in the stomach, duodenum, and jejunum, alterations of bowel absorption may lead to malabsorption of this vital mineral. Therefore, gastric bypass surgery, gastrectomy, and short-bowel syndrome are common etiological factors of copper deficiencies [15,16]. Copper deficiency results in neuropathy in 10% to 20% of patients after gastric bypass surgery [5]. Gastric bypass surgery reduces net copper absorption by lowering food exposure to gastric acid, which is needed to free the copper from organic complexes and ligands. Apart from that, gastric bypass also reduces net copper absorption by excluding the duodenum and proximal jejunum, which are the main sites for its absorption [17].

The mainstay of treatment for NON is supplementation therapy [7]. Therefore, it is crucial to identify which nutrients are deficient, and it is important to take note that many patients have multiple micronutrient deficiencies. A multidisciplinary approach to treating patients with NON is required for optimal management, which includes ophthalmology, gastroenterology, clinical psychology, clinical biochemistry, dietitians, and other specialists. Besides supplementation therapy, revision surgery can serve as an alternative treatment in cases of severe nutritional deficiency post-BS [18,19,20]. Similarly, in our case, the patient underwent revision of the gastric bypass to normal anatomy. Several factors lead to poor nutritional status in post-BS patients. Lack of adequate nutritional supplementation, together with restricted energy intake with higher protein intake, can lead to malnutrition. Apart from that, altered anatomy and physiology of the gastrointestinal tract secondary to BS, and changes in the gut microbiota are also factors.

The prognosis of NON depends on the severity and the duration between the first signs of NON and the time of micronutrient supplementation. In most cases, there will be complete visual recovery; however, if the NON is chronic, optic atrophy will occur [7]. Apart from that, NON can also lead to permanent visual loss if it is diagnosed too late and/or left untreated. In a case reported by Chacko et al. [8], a female patient was diagnosed with NON secondary to folic acid and vitamin B complex deficiencies three years following BS. Despite being treated with vitamin supplementation, there was no improvement in the visual loss, most likely because the diagnosis was made too late. Chronic, uncompensated, or untreated deficiency will eventually lead to persistent and irreversible destruction of the optic nerve fibers [1], which highlights the critical need for urgent reversal of the malnutrition in these patients upon clinical diagnosis. Our patient was indeed lucky that her eye condition improved, although the vision improvement significantly lagged behind the restoration of micronutrient levels, and decreased central vision sensitivity persisted even to her last follow-up, 18 months later.

In this case, there are a few limitations. Firstly, the thiamine (B1) and B6 levels. These two B vitamin levels were ordered however the results could not be traced. It is important to identify all the possible micronutrients deficiency that could lead to NON in order to aid in the diagnosis and treatment. Secondly, there is the lack of a multifocal ERG in our centre. This test could have been useful for detecting subclinical macular dysfunction.

## Conclusions

NON is an under-recognized and underdiagnosed cause of optic neuropathy. Considering the increasing prevalence of morbid obesity worldwide and growing numbers of BS performed, the incidence of NON is likely to increase. Therefore, all persons subjected to such surgeries should be counselled on ocular morbidity that can occur, and supplementation with vitamins and microelements should be commenced immediately. Furthermore, it is also important to identify the disease in its early phase and to correct the deficiencies as soon as possible with supplementation therapy and/or revision surgery. It is also of importance to note that early micronutrient supplementation may not always lead to full functional recovery. Hence, early detection of the disease and long-term follow-up are crucial in patients undergoing bariatric surgery.
